# Molecular Prevalence and Phylogeny of Tick-Borne Viruses in Meat and Dairy Products in the Republic of Korea

**DOI:** 10.1155/2024/8131727

**Published:** 2024-01-29

**Authors:** Yeeun Seo, Md. Iqbal Hossain, Zhaoqi Wang, Daseul Yeo, Soontag Jung, Seoyoung Woo, Yuan Zhang, Min Suk Rhee, Changsun Choi

**Affiliations:** ^1^Department of Food and Nutrition, College of Biotechnology and Natural Resources, Chung-Ang University, Anseong, Gyeonggi-do 17546, Republic of Korea; ^2^Department of Biotechnology, College of Life Sciences and Biotechnology, Korea University, Seoul 02841, Republic of Korea

## Abstract

Tick-borne virus detection in livestock and slaughterhouse animals has recently surged in the United States and Europe. Although cases of patients with tick-borne illnesses have been reported in Korea, food contamination from tick-borne viruses has yet to be investigated. Therefore, this study investigated severe fever with thrombocytopenia syndrome virus (SFTSV), tick-borne encephalitis virus (TBEV), and Crimean–Congo hemorrhagic fever virus (CCHFV) prevalence in meat and dairy products. A total of 628 products were collected from a Korean retail market during 2021–2022, including 195 beef, 130 goats, 90 lambs, 61 pork, 50 chicken, and 38 commercial cheese samples. In addition, 64 raw cow milk samples were collected from a ranch in Anseong-si, Gyeonggi-do, from 2021 to 2022. Real-time reverse transcription-polymerase chain reaction (RT-PCR), nested reverse transcription polymerase chain reaction (RT-nPCR), virus cultivation, and sequence analysis were conducted. SFTSV was detected in 1.53% (3/195) beef and 0.76% (1/130) goat meat samples with a low Ct value titer from 33.18 to 38.60. In contrast, SFTSV was neither detected in lamb, pork, chicken, raw milk, or cheese samples nor were TBEV and CCHFV detected in any of the tested samples. Although no existing cases or studies have indicated SFTSV transmittance through food, this study confirmed SFTSV genotype B RNA in SFTSV-positive meat samples. Therefore, monitoring for and evaluating SFTSV-contaminated meat products must be investigated in future studies.

## 1. Introduction

Ticks are prominent vectors for pathogen and viral transmission, and viruses carried by ticks are known as tick-borne viruses (TBVs). TBVs are emerging pathogens becoming increasingly prevalent in new hosts and geographic locations [1]. Ticks' impact on virus transmission has long been established, and new TBVs are continuously detected as known viruses spread to new geographic locations [2]. Numerous TBVs have since been discovered, many of which cause diseases in animals and humans, with some linked to severe outbreaks. However, preparation for emerging vector-borne diseases, such as tick-borne illnesses, is severely lacking. Therefore, preemptive research on TBV emergence is crucial. Despite existing cases of food poisoning from tick-borne encephalitis virus (TBEV) and Crimean–Congo haemorrhagic fever virus (CCHFV), food studies on TBVs are scarce [3, 4].

Severe fever with thrombocytopenia syndrome virus (SFTSV) belongs to the Bunyaviridae family and *Phlebovirus* genus. SFTSV is a newly discovered bunyavirus with high pathogenicity in humans. The viral gene comprises three segments: L encodes an RNA-dependent RNA polymerase, M encodes a glycoprotein, and S encodes nonstructural and nucleocapsid proteins (NP) [5]. SFTSV is transmitted through tick (*Haemaphysalis longicornis*) bites or contact with tissue or blood of infected livestock or human patients [6]. SFTS is associated with fever, vomiting, diarrhea, and enlarged lymph nodes, and severe cases may beget death. SFTSV was first reported in China in 2009, and cases are escalating yearly in Asian countries such as Myanmar, China, Korea, and Japan [7]. In August 2012, the first patient who died from high fever, severe thrombocytopenia, and multiple growth failures were reported in Korea [8]. In South Korea, 1,332 cases and 281 deaths have been reported from 2013 to 2020 [9]. In addition, approximately 170 SFTSV cases are annually reported in South Korea [10], where it is managed as a communicable disease.

TBEV is an enveloped, single-stranded RNA virus of the family Flaviviridae and genus *Flavivirus*. TBEV is transmitted to humans through *Ixodes* genus tick bites or by consuming raw milk, unpasteurized milk, or milk products from cows, goats, and sheep infected by tick bites. Several TBEV foodborne outbreaks were reported in the EU, and TBEV infections have surged in the EU within the last decade [3]. For example, TBEV was detected in 5% of cow milk samples in Norway [11], and a case in Slovakia originated from unpasteurized cheese consumption [12]. However, based on systematic review and meta-analysis of European data, most human cases from consuming raw milk products are associated with goat milk [13, 14].

CCHFV is a TBV from the Nairoviridae family and *Orthonairovirus* genus [15]. Because of its vector's extensive geographic distribution (ticks from the genus *Hyalomma*), CCHF is the most widespread tick-borne disease affecting humans [16, 17]. CCHFV is transmitted through tick bites, tick crushing, or contact with tissue or blood from infected livestock or human patients [18]. In addition, some food poisoning cases are prompted by eating frozen meat contaminated with CCHFV [4]. Clinical symptoms are characterized by bleeding, fever, muscle pain, headache, myalgia, and dizziness but vary between regions [19, 20].

Some viruses have recently been isolated from food products and regarded as emerging foodborne pathogens due to possible human transmission through the food chain. Consuming contaminated foods (unpasteurized milk and milk or meat products) from infected animals can potentially spread zoonotic viruses like the Nipah virus, Ebola virus, avian influenza virus, Aichi virus, TBEV, and coronaviruses (SARS-CoV-1, SARS-CoV-2, and MERS-CoV) to humans, effectuating gastroenteritis [21–23]. Moreover, the emergence and re-emergence of some TBV-related epidemics or sporadic cases have also been reported in areas with a history of diseases and new geographic regions. These observations are a continuous reminder that TBVs have always been a significant global public health concern. Although many studies have been conducted on viruses from ticks, few studies have investigated TBVs in food. Therefore, this study compared detection methods for three TBVs (SFTSV, TBEV, and CCHFV) in food and investigated virus prevalence in meat and dairy products.

## 2. Materials and Methods

### 2.1. Tick-Borne Virus Detection through Real-Time RT-PCR

TBV quantitation was achieved through real-time reverse transcription-polymerase chain reaction (RT-PCR). Real-time RT-PCR was performed in a 25 *μ*L reaction volume, consisting of 5 *μ*L RNA, 12.5 *μ*L QuantiTect Probe RT-PCR Master Mix (Qiagen, Hilden, Germany), 0.25 *μ*L RT mix (RevertAid Reverse Transcriptase mix), 500 nM of each primer (final concentration), 250 nM probe, and diethylpyrocarbonate (DEPC)-treated water up to a total reaction volume of 25 *μ*L. Table S1 and Table 1 show sequence information for selecting primers and probes for SFTSV, TBEV, and CCHFV detection. Real-time RT-PCR was performed with the CFX96™ Real-Time PCR system (Bio-Rad Laboratories, USA).

The virus stock, an animal-derived SFTSV-positive control (0.025% formaldehyde solution inactivated), was obtained from the Animal and Plant Quarantine Agency (Hyeoksin, Gimcheon, Republic of Korea), and Dog/Korea/JJCB01/2020 (JJCB01/2020) was isolated from serum samples of clinically ill dog companions in October 2018. The reference TBEV RNA (TBEV European subtype prototypic strain Neudoerfl, GenBank accession no. U27495) was collected as positive control from the Korea Centers for Disease Control and Prevention (KCDC). A CCHFV-positive control was synthesized and used as synthetic RNA, which was produced according to GenBank sequences (MN866200 and KC867274.1 GenBank accession number). The nucleoprotein encoding region was selected as set A (112 bp), set B (115 bp), and set C (115 bp), including the selected primer region. Ultramer RNA oligos were formulated at a 4 nmole concentration and purified through a standard desalting method. The synthetic CCHFV RNA 1 sequence was 5ʹUCUCAAAGAAACACGUGCCGCUUACGCCCACAGUGUUCUCUUGAGUGUUAGCAAAAUGGAGAGGUGAAUAACAAAGAUGAGAUGAACAAGUGGUUUGAAGAGUUCAAAAAGG-3ʹ, and the synthetic CCHFV RNA 2 sequence was 5ʹCAAGGGGUACCAAGAAAAUGAAGAAGGCAGCUCUUCGCCGAUGAUUCUUUCCAAACAGGAUCUACAUGCACCCUGCUGUGUUACAGGUGUCUGCUUUGGAACAAUCCCUGUGGC-3ʹ. Primer–probe set A was tested for synthetic RNA 1, and primer–probe sets B and C were tested for synthetic RNA 2.

### 2.2. Sample Preparation and Nucleic Acid Extraction

A total of 628 samples (195 beef, 130 goats, 90 lambs, 61 pork, 50 chicken, 64 raw cow milk, and 38 commercial cheese) were collected from the online market and different farms all over the Republic of Korea from 2021 to 2022 as follows: imported: 20 beef, 20 goats, and 20 lambs; and domestic: *Haenam*—25 beef, 20 goats, 10 lambs, 10 pork, and eight chicken; *Yangpyeong*—35 beef, 10 goats, 10 lambs, 10 pork, and 10 chicken; *Geochang*—25 beef, 20 goats, 15 lambs, 11 pork, and seven chicken; *Hoengseong*—30 beef, 20 goats, 10 lambs, 10 pork, and 10 chicken; *Boseong*—30 beef, 20 goats, 15 lambs, 10 pork, and seven chicken; *Andong*—30 beef, 20 goats, 10 lambs, 10 pork, and eight chicken; 38 commercial cheese (18 processed and 20 naturals); and *Anseong-si*—64 raw cow milk.

Meat sample preparation followed the “Food Poisoning Cause Investigation Test Method (2022)” *by the* Ministry of Food and Drug Safety (MFDS). Specimens were cut into 5 g pieces using sterile scissors and tweezers, then chopped and homogenized. Murine norovirus (MNV-1) was inoculated throughout the sample as a process control. Next, 45 mL of PBS (pH 7.4) was added, and the sample was homogenized for 5 min with a vortexer. Afterward, the sample was transferred to a 50 mL tube for centrifugation (10,000x *g* for 30 min at 4°C), and the suspended matter was separated using the stomacher bag filter. The supernatant was then placed in a Vivaspin 20 UF membrane filter with 15 mL of desorption solution and concentrated at 8,000x *g* for 30 min. After transferring the desorption solution to a new Vivaspin 20 UF membrane filter, the sample was again concentrated at 8,000x *g* for 30 min. Finally, the recovered aqueous phase (500 *µ*L of the final solution) containing virus particles was used for RNA extraction.

The milk sample was prepared [27], and virus recovery was tested. Briefly, 10 mL of raw milk was placed in a 15 mL conical tube and inoculated with MNV as a process control. The sample was then degreased through centrifugation (6,000x *g* for 10 min), and the skim milk was transferred from the bottom of the tube to another with a syringe. The recovered aqueous phase (500 *µ*L of the final solution) containing virus particles was used for RNA extraction.

The cheese sample was prepared [28], and virus recovery was tested. Cheese samples weighing 6 g were inoculated with MNV as a process control and placed in a filter bag. Cold 2% trisodium citrate 1 : 10 (w/v) was then supplemented and homogenized with a stomacher for 1 min. The supernatant was moved to a 50 mL tube and centrifuged (300x *g* for 10 min) at 4°C, then transferred to a new 50 mL tube and centrifuged (5,000x *g* for 45 min) at 4°C. Next, the supernatant was diluted 1 : 5 with cold SM buffer (200 mM NaCl, 10 mM MgSO_4_, and 50 mM Tris, pH 7.5). Then, 10% polyethylene glycol (PEG) 8000 was added to the solution and stored overnight at 4°C for virus particle precipitation. The solution was then centrifuged at 4°C at 6,000x *g* for 1 hr, discarded, and the pellet was resuspended with 2 mL of cold SM buffer. Next, 1 mL of resuspension solution and 1 mL of chloroform were vortexed with the solution for 1 min and centrifuged (15,000x *g* for 5 min) at 4°C. The recovered aqueous phase (500 *µ*L of the final solution) containing virus particles was used for RNA extraction.

Next, 10 *µ*L of MNV-1 was inoculated at a concentration of 2.0 × 10^5^ plaque-forming units (PFU)/mL as a process control for virus recovery rate experiments; process control detection was based on the MNV-1 *VP1* gene. Oligonucleotide sequence information and reaction conditions used for RT-PCR to detect the process control (MNV-1) are listed below. The primer sequences used were as follows: forward primer: 5ʹ- ACGCTCAGCAGTCTTTGTGA-3ʹ, reverse primer: 5ʹ-CTGGCCTCAGAGCCATTG-3ʹ, and probe: 5ʹ-FAM-CGCTGCGCCATCACTCATCC-BHQ-3ʹ. Reverse transcription was performed at 50°C for 30 min, and preheating was performed at 95°C for 15 min. Thermocycling conditions included 45 cycles of an initial denaturation at 95°C for 15 s and annealing and extension at 60°C for 45 s.

The final recovered aqueous phase (500 *µ*L of the final solution) from the previously described meat and dairy product preparation was taken, and viral RNA was extracted using the RNeasy Mini Kit (Qiagen, Germany) following the manufacturer's instructions. Extracted RNA was stored at −80°C until real-time RT-PCR or RT-nPCR was performed.

### 2.3. RT-nPCR and Cloning

Real-time RT-PCR-positive samples were used for RT-nPCR [24]. RT-nPCR was performed with the T100 Thermal Cycler (Bio-Rad Laboratories, USA) using a One-Step RT-PCR Kit (Bioneer, Daejeon, Korea) following the manufacturer's instructions. The primer sequences used are as follows: primary forward primer: CATCATTGTCTTTGCCCTGA, primary reverse primer: TTCAGCCACTTCACCCGRA, secondary forward primer: ATCATCAAGGCATCAGGGAA, and secondary reverse primer: AGAAGACAGAGTTCACAGCA [29]. RT-nPCR was completed as follows: reverse transcription (RT) at 50°C for 30 min, DNA polymerase activation at 94°C for 5 min, 35 cycles of initial denaturation at 94°C for 30 s, annealing at 60°C for 30 s, an extension at 72°C for 1 min, and a final extension at 72°C for 5 min. The primary nested PCR products were used as templates for secondary nested PCR reactions performed using *Top* DNA polymerase (Bioneer, Daejeon, Korea). Initial denaturation was performed at 94°C for 5 min. The thermocycling conditions comprised 35 cycles of denaturation at 94°C for 20 s, annealing at 60°C for 30 s, extension at 72°C for 1 min, and a final extension at 72°C for 7 min. RT-nPCR products were visualized on a 1.0% agarose gel stained with 0.1 *μ*L/mL of UltraPure™ Ethidium Bromide (Invitrogen, USA) using a horizontal electrophoresis system at 100 V for 90 min (Bio-Rad Laboratories, USA).

Positive sample cloning was achieved with the pDyne TA-Blunt V2 kit (Dyne Bio Inc., Republic of Korea) following the manufacturer's instructions. Insertion was completed by reacting the amplified PCR product with pDyne TA-Blunt V2 (Dyne, Korea) at room temperature for 5 min. The transformation was performed with DH5*α* chemically competent *E. coli* iced for 30 min. After heat-shocking the sample at 42°C for 30 s, it was immediately transferred to ice and stabilized for 2 min. Then, 400 *μ*L of super optimal broth with catabolite repression (SOC) media was added, and cells were incubated for 1 hr at 37°C using a shaker. Next, 500 *µ*L of the culture solution was applied to a lysogeny broth (LB) medium containing ampicillin and incubated overnight. The total reaction volume contained 10 *μ*L of Dyne 2x DyeMIX-High, 5 pmol/*μ*L of M13 forward (5ʹ-CAGGAAACAGCTATGAC-3ʹ) and reverse (5ʹ-GTAAAACGACGGCCAG-3ʹ) primers, 1 *μ*L of the suspended colony, and DEPC-treated water up to 20 *μ*L. Cycling conditions were as follows: initial denaturation at 95°C for 2 min, 30 cycles of denaturation at 95°C for 30 s, annealing at 55°C for 30 s, extension at 72°C for 1 min, and a final extension at 72°C for 7 min. Cloned PCR products were visualized using a 1.5% agarose gel stained with 0.1 *μ*L/mL of UltraPure™ Ethidium Bromide, and the sequences were verified through DNA sequencing.

### 2.4. Sequencing and Phylogenetic Analysis

Cloned products were sequenced in both directions with real-time RT-PCR primers through capillary electrophoresis using the BigDye® Terminator v3.1 Cycle Sequencing Kit (Thermo Fisher Scientific, USA) on a SeqStudio Genetic Analyzer (Thermo Fisher Scientific, USA). The sequence data were edited using the SeqMan program (DNASTAR, USA) and analyzed with different viral reference genotype sequences from the National Center for Biotechnology Information Basic Local Alignment Search Tool. Sequences were also phylogenetically analyzed with the SeqMan program (DNASTAR, USA).

Four sequences were aligned based on the Kimura 2-parameter model to confirm SFTSV-positive samples' phylogenetic status. The optimal base substitution model was then calculated, and the maximum likelihood phylogenetic tree was constructed using MEGA X software [30] and evaluated by the bootstrap test with 1,000 replications. Tree percentages with clustered related taxa are displayed next to the branches [31].

### 2.5. Hematoxylin and Eosin (H&E) Staining

For histopathologic staining, frozen muscle tissues were fixed through immersion in 10% formalin at room temperature, followed by cross-sectioning. Fixed tissues were successively dehydrated by rinsing with ethanol at increasing concentrations and cleaned with xylene. The sections were then paraffin-embedded at a 5 *μ*m thickness. Muscle sections were then stained with H&E to study virus-induced pathology. Paraffin-embedded sections were deparaffinized through immersion in xylene and rehydrated with descending ethanol concentrations. After a 5 min hematoxylin incubation, slides were washed with 1% lithium carbonate for 10 min. Next, slides were rinsed with water, stained with 1% eosin for 30 s, and dehydrated with ascending ethanol concentrations [32]. The H&E-stained section was then fixed with permanent mounting medium (VectaMount; Vector Laboratories, USA) and examined under a microscope at 400x magnification (Leica DM2500, USA).

### 2.6. Virus Cultivation

The 96-well plates were seeded with 1,000 Vero cells (ATCC CCL-81) per well at a passage number of 40 and incubated for 24 hr before virus infection. The virus-concentrated supernatant from the SFTSV-positive meat sample was filtered through a 0.45 *μ*m syringe filter. The supernatant was diluted in DMEM supplemented with 2% FBS and 10 *µ*L/mL antibiotic–antimycotic and inoculated at 100 *µ*L per well. The inoculum (virus-concentrated supernatant) was removed after 24 hr, and each well was filled with 100 *µ*L of DMEM containing 2% FBS and incubated to observe cytopathic effects (CPE) 5 days postinoculation [33].

## 3. Results

### 3.1. Comparison of Primers and Probes Used for Tick-Borne Virus Detection

#### 3.1.1. Real-Time RT-PCR Results of Primer–Probe Sets for SFTSV Detection

Sensitivity evaluation was performed through serial 10-fold dilutions of the reference SFTSV-positive stock.  Table 2 conveys the sensitivity results for each S, M, and L segment of the primer–probe sets targeting the SFTSV YG1 [34] and SFTSV HB21 strains [29]. Regarding the YG1 strain, the S and M segments presented 37.01 and 38.11 cycle thresholds (Ct) at 1.00 log TCID_50_/reaction, respectively; the L segment showed a 33.49 Ct at 2.00 log TCID_50_/reaction. Regarding the HB21 strain, the S segment displayed a 40.29 Ct at 3.00 log TCID_50_/reaction, whereas the M and L segments indicated 39.27 and 37.93 Ct values at 4.00 log TCID_50_/reaction, respectively. Notably, the M and L segments were better amplified in HB21, and the S segment was better amplified in YG1.

Next, we selected a primer–probe set that targets the YG1 strain's S segment. It is a specific real-time RT-PCR primer–probe set targeted the NP based on the SFTSV YG1 (a Japanese strain) nucleotide sequence. When detecting SFTSV using YG1 strain primer–probe set targeting the S segment, it showed 100.0% specificity, 0.0% false positive rate, and 0.0% false negative rate. SFTSV primer specificities were tested using various viruses as negative controls: Coxsackievirus A2, Norovirus GI, Norovirus GII, Human bocavirus, Adenovirus, Influenza A virus H1N1, Echovirus 25, Rotavirus, and Murine norovirus. Since the controls were not detected, the YG1 real-time RT-PCR set targeting SFTSV's S segment was determined to have specificity for the virus.

#### 3.1.2. Nested RT-PCR (RT-nPCR) Results of the SFTSV Detection Primer–Probe Sets

Sensitivity was evaluated using serial 10-fold dilutions of the reference SFTSV-positive stock. After testing the sensitivity of primers using RT-nPCR, the ability to detect up to 1.00 log TCID_50_/reaction virus titers was confirmed (data not shown). In addition, during the RT-nPCR specificity test of SFTSV primers with various viruses, Coxsackievirus A2, Norovirus GI, Norovirus GII, Human bocavirus, Adenovirus, Influenza A virus H1N1, Echovirus 25, Rotavirus, and Murine norovirus were not detected (data not shown). Therefore, the YG1 RT-nPCR set for SFTSV's S segment was determined to have specificity for the virus.

#### 3.1.3. Real-Time RT-PCR Results of Primer–Probe Sets for TBEV Detection

Rather than the NS5 protein that encodes the methyltransferase for RNA capping, the NS1 protein that encodes mRNA was a more suitable method for TBEV detection in food. Sensitivity was evaluated using serial 10-fold dilutions of the reference TBEV RNA stock. Real-time RT-PCR primer–probe sets targeting TBEV's NS1 indicated a 37.39 Ct at a −3 serial dilution from the original RNA stock (Table 2). Conversely, the real-time RT-PCR primer–probe set targeting TBEV's NS5 exhibited the same detection as NS1 but with a higher 39.92 Ct. Therefore, the real-time RT-PCR primer–probe sets for detecting TBEV were designed to target NS1. When detecting TBEV with a primer–probe set targeting NS1, 100.0% specificity, 0.0% false positive rate, and 0.0% false negative rate were achieved.

#### 3.1.4. Real-Time RT-PCR Results of Primer–Probe Sets for CCHFV Detection

Sensitivity was evaluated using serial 10-fold dilutions of the synthetic reference CCHFV RNA (Table 2). The primer–probe sets' detection limit in real-time RT-PCR for CCHFV detection was as follows: primer–probe set A detected 10^3^ genome copies/reaction with a 37.82 Ct, primer–probe set B detected 10^3^ genome copies/reaction with a 37.90 Ct, and primer–probe set C was not the most sensitive and detected 10^7^ genome copies/reaction with a 43.80 Ct (Table 2). Although primer–probe sets A and B had similar sensitivities, set B was used for CCHFV detection as set A had excessive signal noise. When detecting CCHFV using primer–probe set B, 100.0% specificity, 0.0% false positive rate, and 0.0% false negative rate were achieved.

#### 3.1.5. Virus Recovery in Meat, Milk, and Cheese Products

The MFDS meat pretreatment method confirmed a 40.79 ± 0.25% MNV-1 process control recovery rate in meat; the sample preparation method established 21.73% ± 2.50% and 6.83% ± 1.09% MNV-1 process control recovery rates in milk and cheese, respectively. MNV-1 (process control) extraction efficacy was at least 6.83%, which still exceeds the minimal 1% recovery rate obligatory for successful food sample analysis [35]. The recovery method's efficiency for each sample was calculated based on the quantified copies through RT-PCR as follows:

(1) $$\begin{array}{c} \text{Recovery efficiency}\left(\%\right)=\frac{\text{Total viral recovery RNA genome copies}}{\text{Total viral seeding RNA genome copies}}\times 100. \end{array}$$

#### 3.1.6. TBV Prevalence in Meat and Dairy Products

TBV prevalence was based on real-time RT-PCR detection (Table 3), and all meat products originated from Korea. SFTSV was identified in 1.53% (3/195) of beef and 0.76% (1/80) of goat samples. Additionally, SFTSV was detected in meat samples with a 33.18–38.60 Ct value. Lambs, pork, chicken, raw milk, and cheese samples were SFTSV negative. Notably, TBEV and CCHFV were not detected in any experimental samples. Slaughter month, detection section, origin, and SFTSV Cts detected in meat products are organized in Table 4.

#### 3.1.7. Sequencing and Phylogenetic Analysis

Scientists are gradually recognizing the necessity for a confirmation step in detection assays, with cloning and sequencing as choice methods. However, cloning and sequencing viruses in food and environmental samples determined as positive through real-time RT-PCR can present challenges, mainly as viruses are often present in samples at low levels. The food matrix contains various PCR inhibitors, such as metal ions and food-derived nucleic acids. It is frequently demanded that nested PCR approaches have superior detection limits for detecting low levels of virus contamination than single amplifications. This study completed a controlled comparison of two detection assays widely used to screen food samples for SFTSV. The assays chosen for comparison were a one-step real-time RT-PCR and a nested real-time PCR, as both assays have broad specificity across SFTSV. Four SFTSV-positive meat samples were identified through real-time RT-PCR but not RT-nPCR; therefore, the sequences were confirmed through short-length real-time RT-PCR not long-length RT-nPCR. The sequences were deposited in GenBank, and the accession numbers are as follows: OP169694 (CAU210252), OP169695 (CAU210319), OP169696 (CAU210324), and OP169697 (CAU210398).

**Figure 1:**
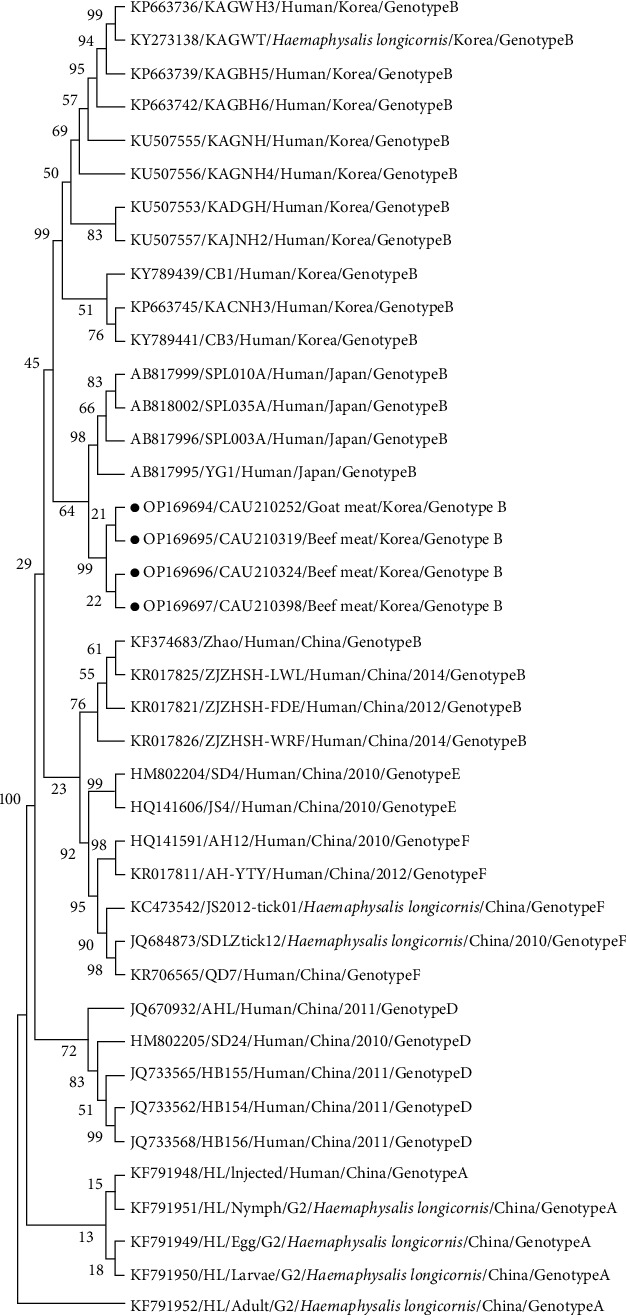
Phylogenetic tree of the SFTSV nucleocapsid (NP) region. Black label (solid circles) represents positive samples isolated from meat products. Numbers near each node represent percentage value.

Next, we phylogenetically analyzed the S-segment viral sequences of four SFTSV-positive samples to understand the relationship between SFTSV strains. Based on the SFTSV NP gene's 137 bp sequence, a phylogenetic analysis of SFTSV was performed with the following reference genotype strains: A (five strains), B (19 strains), D (five strains), E (two strains), and F (five strains) (Figure 1). SFTSV's S segment belonged to genotype B with a 99% bootstrap value (1,000 replicates). The four virus sequences data obtained were 100% consistent and achieved 100% homology with genotype B strains (KF374683, KR017825, KR017826, KR017821, KU507553, and KU507553). All sequences indicated a very high nucleotide identity (∼99.3%) with previously isolated reference genotype A strains (KF791951, KF791952, KF791950, KF791949, and KF791948). The strains also exhibited 97.1%–98.5% homology with the genotype D strains (JQ733562, JQ733568, JQ733565, JQ684873, and HM802205). Furthermore, genotype E and F strains (HM802204, HQ141606, HQ141591, KR017811, KC473542, JQ684873, and KR706565) presented a 97.8%–98.5% homology.

#### 3.1.8. Hematoxylin and Eosin (H&E) Staining in Muscle Tissue

H&E slides for all SFTSV-positive meat samples were prepared, and histopathological changes were evaluated by a veterinary pathologist (the corresponding author is a veterinary pathologist). As with CAU210323 (an SFTSV-negative sample), inflammation and muscle tissue damage were not identified. When mild lymphocytic infiltration was observed in SFTSV-positive beef meat samples (CAU210319), it was not a pathognomonic lesion. However, an SFTSV-specific positive signal was not found through immunohistochemistry (data not shown).

#### 3.1.9. Virus Cultivation

SFTSV was cultivated from meat homogenates *in vitro*. Vero cells were infected with SFTSV isolates from samples, including CAU210252, CAU210319, CAU210324, and CAU210398. Two (CAU210252 and CAU210324) isolates displayed morphologic changes (increased round fused cells in Vero cells) compared to the negative control. However, morphologic changes (increased round fused cells) were not indicated for CPE, as CPEs were not evident or observed microscopically. Therefore, SFTSV cultivation could not be confirmed based on CPE absence.

## 4. Discussion

This study monitored for SFTSV in meat products, confirming three SFTSV-positive (1.53%) beef samples and one SFTSV-positive (0.76%) goat sample through real-time RT-PCR. TBEV and CCHFV were not detected through real-time RT-PCR in meat or dairy samples. Meat samples deemed SFTSV-positive via real-time RT-PCR were further tested with RT-nPCR. However, all four SFTSV-positive samples did not contain viruses detectable by RT-nPCR. This finding is because only small meat product samples were used in the test; thus, there were not enough virus concentrations in the food to support further testing.

So far, SFTSV has only been reported in East Asia (Republic of Korea, Japan, and China) [36]. Although SFTSV and TBEV have been identified in Korea, there are only existing cases of SFTSV [37]. Furthermore, no CCHFV patient reports exist as it has not been introduced in Korea [38]. SFTSV has been found in Korea, but it is undetectable due to the low circulating cases. SFTSV has infected many ticks and humans in several Asian regions, including Korea, but no food-borne cases have been reported [1]. However, TBEV and CCHFV infections from consuming raw milk or meat suggest that tick-borne diseases can also be transmitted through food intake [39, 40].

It is more challenging for a virus to spread across geographic barriers abroad than on the same continent, but it can circulate through trade and infected meat product distributions. As with any food consumption, prioritizing food safety and properly handling raw food is crucial for minimizing risks for foodborne illnesses. Thus, heated food is recommended to encourage safe consumption. The KCDC manages CCHFV as a Group 1 legal infectious disease and TBEV and SFTSV as Group 3 legal infectious diseases. Therefore, the risk of contracting the disease through virus-infected meat consumption is low. This study is the first to confirm tick-borne virus contamination in meat samples collected from Korean retail markets.

This study incorporated gene targeting to amplify and sequence the S segment. The N protein encoded from the S segment acted as a scaffold for viral packing, was highly immunogenic against the *Phlebovirus* genus, and posed as a prominent antigen [41]. In addition, a quantitative real-time RT-PCR assay detecting SFTSV viral RNA in meat and dairy products was developed and evaluated. Assay sensitivity and specificity for detecting SFTSV viral RNA were determined as reliable. The assay technique detected SFTSV's L, M, and S segments simultaneously. A multiple-gene detection test was chosen due to insufficient information on the novel bunyavirus, namely each segment's gene mutation frequency or possibility.

SFTSV's L, M, and S segments were selected as target genes to increase test sensitivity and reduce potential mismatch from viral gene sequence mutations. Multiple primer–probe sets considerably increased detection efficiency in prior experiments [42, 43]. This multiplex strategy enables mutation identification in the three SFTSV segments. In our experiments, three primer-probe sets for detecting the L, M, and S genes could be used for a single assay. We recommend a single primer–pair set and a probe designed for the S segment to reduce testing costs, as the SFTSV's S segment was the most conserved gene compared to other viruses in the genus *Phlebovirus*. Based on its practicality, high sensitivity, specificity, throughput capacity, and minimum carryover contamination, the probe-based real-time RT-PCR assay was verified as one of the most suitable assays for the early detection of infectious agents [44].

The intensifying incidence of human SFTSV infection raises concern [5]. SFTSV's A, C, D, E, and F genotypes were prevalent in China, whereas type B was the most prevalent in Korea [45]. Our phylogenetic tree results also proposed a genetic homology with SFTSV's B genotype. Moreover, the SFTSV-identified sequences highly correlated with strains identified in Korea and China through phylogenetic analysis. Our strains and some Korean and Chinese strains clustered together to form a genotype B clade. These results indicate that some viruses in China have been transmitted to Korea multiple times and vice versa. Furthermore, entire genome sequence analysis may be required for a more comprehensive study through whole-genome sequencing rather than virus genotyping with specific genes.

Various tick-borne diseases in Korea depend on the season and region [46]. All SFTSV-positive samples were acquired from animals slaughtered in May and June, coinciding with active tick periods during warm weather [47]. In addition, SFTSV-positive tick pools in nymphs were highest in May [48], and the high SFTS incidence in June is generally attributed to an adult tick population surge, substantial SFTSV carriers [48]. Different vertebrate species are susceptible to SFTSV infection, as evidenced by the high SFTSV prevalence in endemic area animals, such as cats, dogs, birds, and wild boars [49]. In addition, wild animal numbers, such as deer and wild boar, are increasing due to declining hunters and agricultural populations in mountainous areas, and the preferred area for ticks is expanding. These conditions augment direct contact between animals and humans naive to virus-infected ticks [50].

Four SFTS-positive samples were collected from four cities in Korea (Table 4), corroborating existing research that showed that SFTSV was evenly distributed across various regions of Korea [48]. Likewise, the mild inflammatory response associated with SFTSV infection was due to its histopathology [51]. In addition, CPE was not observed in the cell culture, and effective SFTSV cultures were not achieved, suggesting low infection risk through food intake. However, further investigation is needed to understand whether SFTSV-infected ticks that bite animals carry enough viral RNA to effectuate disease through food intake.

One of the most imperative aspects of public health is veterinary public health (VPH), where veterinarians are responsible for protecting the well-being of animals and humans. Veterinary virologists in VPH coordinate research encompassing the viral pathogenesis of diseases in domestic animals, wild animals, and humans. This cooperation is crucial for elucidating how viruses travel and affect human health and populations over time, as well as preparing for novel human diseases. However, veterinarians must be familiar with the disease's prevalence, risk factors, control techniques, and associated costs and benefits to advise farmers on proper disease management. Therefore, ensuring proper hygiene standards before slaughtering animals is necessary to lessen the likelihood of these viruses entering the food chain. In addition, those with a high risk of contracting the disease through direct contact, such as veterinarians and meat industry personnel, should be more aware of methods to prevent direct transmission.

## 5. Conclusions

This study determined the optimal detection method for three TBVs in food, identifying SFTSV-positive samples in meat products. No cases or research have evidenced SFTSV transmittance through food; thus, it is doubtful for someone to become sick from simply consuming meat products infected with tick-borne viruses. Consequently, heating food during handling is sufficient for preventing diseases caused by tick-borne viruses. However, a continuous study on viral transmission from meat should be considered to prevent potential SFTSV transmission through food, as it is a hazardous virus with a high mortality rate. This research will aid in identifying SFTSV-infected animals used as food sources and evaluating possible transmission. In addition, TBEV and CCHFV have not been transmitted through food in Korea, and there are currently no existing cases of human infection throughout the region. Therefore, potential TBEV and CCHFV emergence must be carefully monitored, and further investigation and surveillance are required.

## Figures and Tables

**Table 1:** Selected real-time RT-PCR primer and probe sets for SFTSV, TBEV, and CCHFV detection.

Target	Primers/probes	Oligonucleotide sequence (5ʹ→3ʹ)	Genome position	Amplification conditions	Reference
SFTSV	F1	TGTCAGAGTGGTCCAGGATT	45–64	95°C for 30 min, 95°C for 15 min, repeat 45 cycles of 94°C for 15 s, and 60°C for 60 s	[24]
	R1	ACCTGTCTCCTTCAGCTTCT	162–181		
	P1	FAM-TGGAGTTTGGTGAGCAGCAGC-BHQ	69–86		

TBEV	TBE F	TGGAYTTYAGACAGGAAYCAACACA	2,966–2,990	50°C for 30 min, 95°C for 10 min, repeat 40 cycles of 95°C for 15 s, and 60°C for 45 s	[25]
	TBE R	TCCAGAGACYTYGRTCDGTGTGGA	3,064–3,041		
	TBE probe	FAM-CCCATCACTCCWGTGTCAC-BHQ	3,014–2,996		

CCHFV	RWC F	CAAGGGGTACCAAGAAAATGAAGAAGGC	1,047–1,074	50°C for 20 min, 95°C for 15 min, repeat 44 cycles of 95°C for 10 s, 54°C for 20 s, and 72°C for 15 s	[26]
	RWC R	GCCACAGGGATTGTTCCAAAGCAGAC	1,202–1,227		
	SE01	FAM-ATCTACATGCACCCTGCTGTGTTGACA-BHQ	1,172–1,198		

**Table 2:** Detection limit comparisons for all real-time RT-PCR primer–probe sets for SFTSV, TBEV, and CCHFV detection.

SFTSV					
Target region	Virus titers (TCID_50_/reaction)	Average Ct value	Target region	Virus titers (TCID_50_/reaction)	Average Ct value
YG1 S segment	0.01 log	N/D	HB21 S segment	0.01 log	N/D
	0.10 log	N/D		0.10 log	N/D
	1.00 log	37.01		1.00 log	N/D
	2.00 log	35.14		2.00 log	N/D
	3.00 log	33.76		3.00 log	40.29
	4.00 log	29.11		4.00 log	36.95
	5.00 log	25.18		5.00 log	32.28

YG1 M segment	0.01 log	N/D	HB21 M segment	0.01 log	N/D
	0.10 log	N/D		0.10 log	N/D
	1.00 log	38.11		1.00 log	N/D
	2.00 log	36.54		2.00 log	N/D
	3.00 log	32.7		3.00 log	N/D
	4.00 log	29.62		4.00 log	39.27
	5.00 log	26.29		5.00 log	32.43

YG1 L segment	0.01 log	N/D	HB21 L segment	0.01 log	N/D
	0.10 log	N/D		0.10 log	N/D
	1.00 log	N/D		1.00 log	N/D
	2.00 log	33.49		2.00 log	N/D
	3.00 log	30.87		3.00 log	N/D
	4.00 log	27.62		4.00 log	37.93
	5.00 log	24.27		5.00 log	31.80

TBEV					

Serial dilution from original RNA stock	Average Ct value				
	Target region				
	NS1			NS5	

NC	N/D			N/D	
−5	N/D			N/D	
−4	N/D			N/D	
−3	37.39			39.92	
−2	33.20			34.30	
−1	28.97			29.72	
0 (original RNA stock)	25.73			26.29	

CCHFV					

Genome copies/reaction	Average Ct value				
	Target primer–probe sets				
	Set A		Set B	Set C	

NC	N/D		N/D	N/D	
10^1^	N/D		N/D	N/D	
10^2^	N/D		N/D	N/D	
10^3^	37.82		37.90	N/D	
10^4^	36.94		34.23	N/D	
10^5^	33.18		31.18	N/D	
10^6^	30.18		27.96	N/D	
10^7^	26.24		24.96	43.80	
10^8^	23.78		21.02	39.05	

TCID_50_, 50% tissue culture infectious dose; Ct, cycle threshold; NC, negative control; N/D, negative detection.

**Table 3:** SFTSV, TBEV, and CCHFV detected in food products using real-time RT-PCR.

Sample type	SFTSV	TBEV	CCHFV
Beef	3/195 (1.53%)	0/195 (0.0%)	0/195 (0.0%)
Goat	1/130 (0.76%)	0/130 (0.0%)	0/130 (0.0%)
Lamb	0/90 (0.0%)	0/90 (0.0%)	0/90 (0.0%)
Pork	0/61 (0.0%)	0/61 (0.0%)	0/61 (0.0%)
Chicken	0/50 (0.0%)	0/50 (0.0%)	0/50 (0.0%)
Raw milk	0/64 (0.0%)	0/64 (0.0%)	0/64 (0.0%)
Cheese	0/38 (0.0%)	0/38 (0.0%)	0/38 (0.0%)
Total	4/628 (0.63%)	0/628 (0.0%)	0/628 (0.0%)

**Table 4:** SFTSV detected in food using real-time RT-PCR.

Sample type	Sample number	Slaughter month	Section	Origin	Ct value
Goat	CAU210252	June 2021	Foreshank	Haenam, Jeollanam-do, Korea	33.18
Beef	CAU210319	May 2021	Thin skirt	Hoengseong, Gangwon-do, Korea	36.44
Beef	CAU210324	May 2021	Loin end	Boseong, Jeollanam-do, Korea	37.79
Beef	CAU210398	May 2021	Brisket	Andong, Gyeongsangbuk-do, Korea	38.60

## Data Availability

The datasets presented in this study can be found in online repositories. The names of the repository/repositories and accession number(s) can be found in the article.

## Supplementary Materials

Supplementary Materials Table S1: real-time RT-PCR primer–probe sets for SFTSV, TBEV, and CCHFV.
