# Supplementary material for: Molecular Prevalence and Phylogeny of Tick-Borne Viruses in Meat and Dairy Products in the Republic of Korea
Source: Transbound Emerg Dis. 2024 Jan 29;2024:8131727. doi: 10.1155/2024/8131727 (PMC12016795; doi:10.1155/2024/8131727)
Supplement: Supplementary Materials — Table S1: real-time RT-PCR primer–probe sets for SFTSV, TBEV, and CCHFV. [file 8131727.f1.docx]

## Supplementary Materials:

**Supplementary Table S1. Real-time RT-PCR primer-probe sets for SFTSV, TBEV, and CCHFV.**

**(A) Primer-probe sets for detecting severe fever with thrombocytopenia syndrome virus (SFTSV).**

| **Set** | **Target** | **Primer/probe** | **Oligonucleotide sequence (5’-3’)** | **Genome position** | **Amplification condition** | **Reference** |
| --- | --- | --- | --- | --- | --- | --- |
| **HB21** | **S segment** | S-F | GGGTCCCTGAAGGAGTTGTAAA | 1104-1125 | 50 °C for 30 min, 95 °C for 10 min, repeat 40 cycles of  95 °C for 15 s, and  60 °C for 45 s | [1] |
|  |  | S-R | TGCCTTCACCAAGACTATCAATGT | 1155-1178 |  |  |
|  |  | S-Probe | FAM-TTCTGTCTTGCTGGCTCCGCGC-BHQ | 1127-1148 |  |  |
|  | **M segment** | M-F | AAGAAGTGGCTGTTCATCATTATTG | 1369-1393 |  |  |
|  |  | M-R | GCCTTAAGGACATTGGTGAGTA | 1394-1420 |  |  |
|  |  | M-Probe | FAM-TCATCCTCCTTGGATATGCAGGCCTCA-BHQ | 1424-1445 |  |  |
|  | **L segment** | L-F | 5′-AGTCTAGGTCATCTGATCCGTTYAG-3′ | 3138-3162 |  |  |
|  |  | LR | 5′-TGTAAGTTCGCCCTTTGTCCAT-3′ | 3209-3230 |  |  |
|  |  | L-Probe | FAM-CAATGACAGACGCCTTCCATGGTAATAGGG-BHQ | 3168-3197 |  |  |
| **YG1** | **S segment** | F 1 | TGTCAGAGTGGTCCAGGATT | 45-181 | 95 °C for 15 min, 50 °C for 30 min, repeat 45 cycles of  94 °C for 15 s, and  60 °C for 60 s | [2] |
|  |  | R 1 | ACCTGTCTCCTTCAGCTTCT |  |  |  |
|  |  | P 1 | FAM-TGGAGTTTGGTGAGCAGCAGC-BHQ |  |  |  |
|  | **M segment** | F 2 | GGCAGCTACATGCAGACATA | 3022-3132 |  |  |
|  |  | R 2 | CCTATCACCCCCAGAATCCA |  |  |  |
|  |  | P 2 | FAM-GCCCTGTTTGGCAATGGGCT-BHQ |  |  |  |
|  | **L segment** | F 3 | AACATCCTGGACCTTGCATC | 2537-2659 |  |  |
|  |  | R 3 | CAATGTGGCCATCTTCTCCA |  |  |  |
|  |  | P 3 | FAM-TGGGAGCTCTACTCAGAAGTCCA-BHQ |  |  |  |

**(B) Primer-probe sets for detecting tick-borne encephalitis virus (TBEV).**

| **Set** | **Target** | **Primer/probe** | **Oligonucleotide sequence (5’-3’)** | **Genome position** | **Amplification Condition** | **Reference** |
| --- | --- | --- | --- | --- | --- | --- |
| **1** | **NS1** | TBE F | TGGAYTTYAGACAGGAAYCAACACA | 2966-2990 | 50 °C for 15 min, 95 °C for 2 min, repeat 45 cycles of 95 °C for 15 s, and 60 °C for 60 s | [3] |
|  |  | TBE R | TCCAGAGACYTYGRTCDGTGTGGA | 3064-3041 |  |  |
|  |  | TBE Probe | FAM-CCCATCACTCCWGTGTCAC-BHQ | 3014-2996 |  |  |
| **2** | **NS5** | F-TBE 1 | GGGCGGTTCTTGTTCTCC | 11054-11071 | 37 °C for 30 min, 95 °C for 10 min, repeat 40 cycles of 95 °C for 15 s, and 60 °C for 60 s | [4] |
|  |  | R-TBE 1 | ACACATCACCTCCTTGTCAGACT | 11099-11121 |  |  |
|  |  | TBE-Probe-WT | FAM-TGAGCCACCATCACCCAGACACA-BHQ | 11073-11095 |  |  |

**(C) Primer-probe sets for detecting crimean-congo haemorrhagic fever virus (CCHFV).**

| **Set** | **Target** | **Primer/probe** | **Oligonucleotide sequence (5'-3')** | **Genome position** | **Amplification condition** | **Reference** |
| --- | --- | --- | --- | --- | --- | --- |
| **A** | S segment | CCHF S1 | TCTCAAAGAAACACGTGCC | 1-19 | 50 °C for 10 min, 95 °C for 2 min, repeat 45 cycles of 95 °C for 10 s, and 60 °C for 40 s | [5] |
|  |  | CCHF S122 | CCTTTTTGAACTCTTCAAACC | 102-122 |  |  |
|  |  | CCHF probe | FAM-ACTCAAGGKAACACTGTGGGCGTAAG-BHQ | 21-46 |  |  |
| **B** | S segment | RWCF | CAAGGGGTACCAAGAAAATGAAGAAGGC | 1047–1074 | 50 °C for 30 min, 95 °C for 15 min, repeat 45 cycles of 94 °C for 15 s, and 59 °C for 30 s | [6] |
|  |  | RWCR | GCCACAGGGATTGTTCCAAAGCAGAC | 1202-1227 |  |  |
|  |  | SE01 | FAM-ATCTACATGCACCCTGCTGTGTTGACA-BHQ | 1172-1198 |  |  |
| **C** | S segment | CCReal P1 | TCTTYGCHGATGAYTCHTTYC | 1138-1158 | 42 °C for 30 min, 95 °C for 5 min, repeat 45 cycles of 95 °C for 15 s, and 60 °C for 60 s | [7] |
|  |  | CCReal P2 | GGGATKGTYCCRAAGCA | 1234-1250 |  |  |
|  |  | Probe | FAM-ACASRATCTAYATGCAYCCTGC-BHQ | 1167-1188 |  |  |

1. Y. Sun, M. Liang, J. Qu, C. Jin, Q. F. Zhang, J. Li, X. Jiang, Q. Wang, J. Lu, W. Gu, S. Zhang, C. Li, X. J. Wang, F. Zhan, W. Yao, Z. Bi, S. Wang, and D. Li, “Early diagnosis of novel SFTS bunyavirus infection by quantitative real-time RT-PCR assay,” *Journal of Clinical Virology,* vol. 53, no. 1, pp. 48–53, 2012.

2. T. Yoshikawa, S. Fukushi, H. Tani, A. Fukuma, S. Taniguchi, S. Toda, Y. Shimazu, K. Yano, T. Morimitsu, K. Ando, A. Yoshikawa, M. Kan, N. Kato, T. Motoya, T. Kuzuguchi, Y. Nishino, H. Osako, T. Yumisashi, K. Kida, F. Suzuki, H. Takimoto, H. Kitamoto, K. Maeda, T. Takahashi, T. Yamagishi, K. Oishi, S. Morikawa, M. Saijo, and M. Shimojima, “Sensitive and specific PCR systems for detection of both Chinese and Japanese severe fever with thrombocytopenia syndrome virus strains and prediction of patient survival based on viral load,” *Journal of Clinical Microbiology*, vol. 52, no. 9, pp. 3325–3333, 2014..

3. K. Achazi, A. Nitsche, P. Patel, A. Radonić, O. D. Mantke, and M. Niedrig, “Detection and differentiation of tick-borne encephalitis virus subtypes by a reverse transcription quantitative real-time PCR and pyrosequencing,” *Journal of Virological Methods*, vol. 171, no. 1, pp. 34–39, 2011.

4. M. Schwaiger and P. Cassinotti, “Development of a quantitative real-time RT-PCR assay with internal control for the laboratory detection of tick-borne encephalitis virus (TBEV) RNA,” *Journal of Clinical Virology*, vol. 27, no. 2, pp. 136–145, 2003.

5. B. Atkinson, J. Chamberlain, C. H. Logue, N. Cook, C. Bruce, S. D. Dowall, and R. Hewson, “Development of a real-time RT-PCR assay for the detection of Crimean–Congo hemorrhagic fever virus,” *Vector-Borne and Zoonotic Diseases*, vol. 12, no. 9, pp. 786–793, 2012.

6. R. Wölfel, J. T. Paweska, N. Petersen, A. A. Grobbelaar, P. A. Leman, R. Hewson, M.-C. Georges-Courbot, A. Papa, S. Günther, and C. Drosten, “Virus detection and monitoring of viral load in Crimean–Congo hemorrhagic fever virus patients,” *Emerging Infectious Diseases*, vol. 13, no. 7, pp. 1097–1100, 2007.

7. O. Hekimoglu, N. Ozer, K. Ergunay, and A. Ozkul, “Species distribution and detection of Crimean Congo hemorrhagic fever virus (CCHFV) in field-collected ticks in Ankara province, central Anatolia, Turkey,” *Experimental and Applied Acarology*, vol. 56, no. 1, pp. 75–84, 2012.
